# Comparison of a tube core and Magill forceps for nasotracheal intubation: a randomised controlled trial

**DOI:** 10.1186/s13063-021-05677-9

**Published:** 2021-10-13

**Authors:** Rui Hu, Jing-Yi Niu, Li-Ning Wu, Hao Sun, Peng Sun, Jia-Ying Huang, Jun-Ma Yu

**Affiliations:** 1 Department of Anesthesiology, The Third Affiliated Hospital of Anhui Medical University (The First People’s Hospital of Hefei), Huaihe Road 390, Hefei, 230061 China; 2 Department of Otorhinolaryngology Surgery, The Third Affiliated Hospital of Anhui Medical University (The First People’s Hospital of Hefei), Huaihe Road 390, Hefei, 230061 China

**Keywords:** Anaesthesia, Intubation, Stylet

## Abstract

**Background:**

Magill forceps are frequently used to complete nasotracheal intubation (NTI). We aimed to identify a tube core that could conveniently facilitate the NTI process without using Magill forceps.

**Methods:**

Sixty patients scheduled for oral and maxillofacial surgeries were enrolled in our study and divided into two groups (30 per group) with no differences with regard to demographic data. In the Magill forceps group (Group M), a wire-reinforced endotracheal catheter was inserted into the trachea using Magill forceps. However, in the tube core group (Group T), a tube core bent to the physiological curve of the nasal cavity and lubricated with aseptic paraffin oil was inserted into the endotracheal catheter and was then withdrawn after the endotracheal catheter was advanced through the glottis under direct vision.

**Results:**

All NTIs were completed successfully, and Magill forceps were not used on any patient in Group T. There was a significant difference in total NTI time between the two groups (Group M, 59.7 (5.1) s vs Group T, 52.4 (3.1) s). Mild epistaxis was observed in 6 patients in Group M and 5 patients in Group T (6/30 vs 5/30, respectively). No damage to oral tissue or teeth was observed in either group.

**Conclusions:**

We conclude that using a tube core, consisting of a disposable sterilised stylet, is a convenient choice for NTI.

**Trial registration:**

Patient enrolment was conducted after registration in the Chinese Clinical Trial Registry (www.Chictr.org.cn, ChiCTR190002 7387). This trial was prospectively registered on 11 November 2019.

## Introduction

Nasotracheal intubation (NTI) is widely used in clinical practice for oral and maxillofacial surgeries. In clinical applications, the tip of the nasotracheal tube will course posteriorly into the oesophagus in most cases. Magill forceps have been frequently used to complete the NTI process in several previous studies [1–4]. However, this instrument may cause rupture of the cuff or mucosal injury and even lead to infection [5–7].

**Fig. 1:**
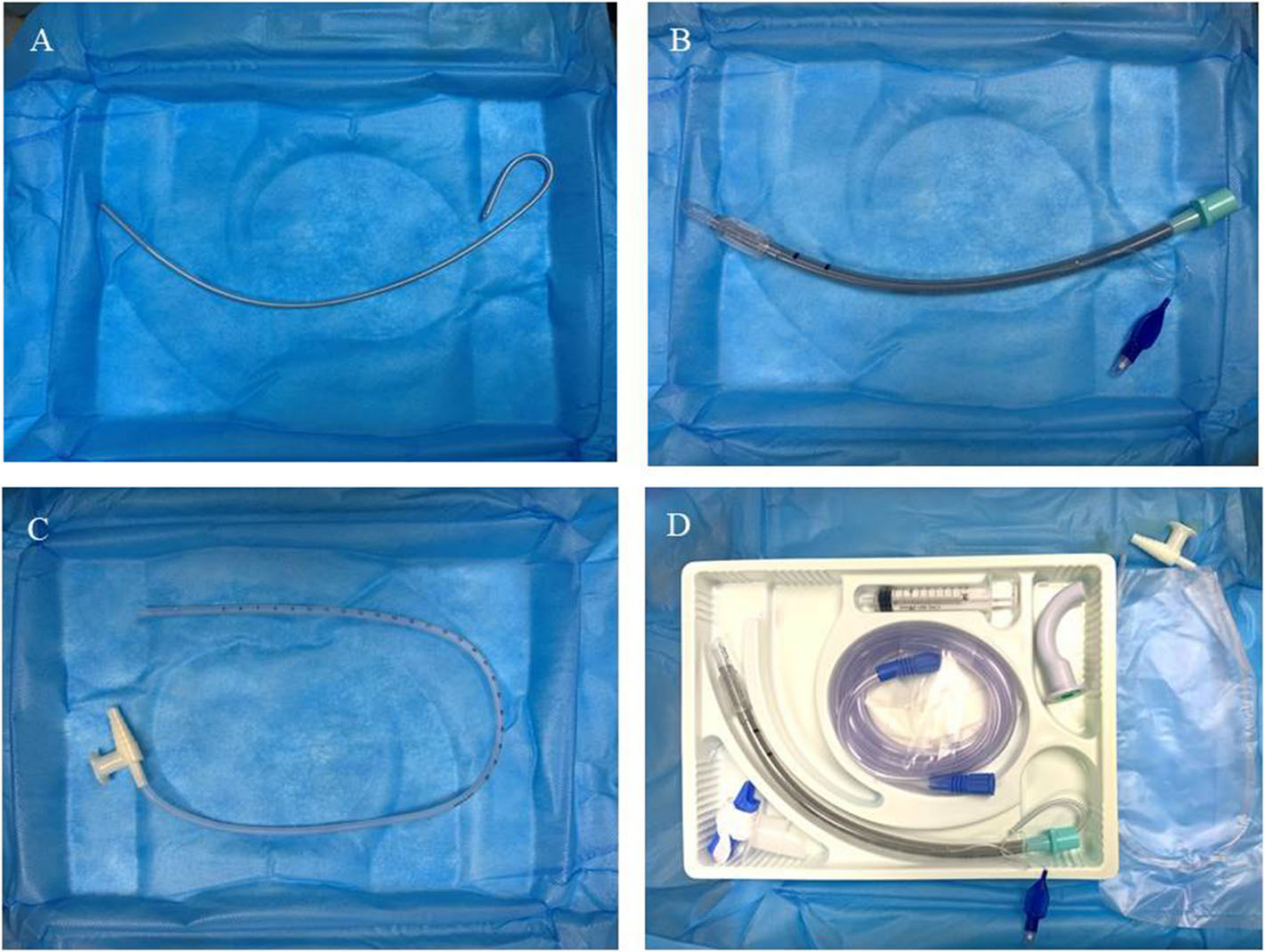
Tube core, a malleable rigid stylet (**A**); wire-reinforced tracheal tube (**B**); aseptic suction catheter (OD - 4.0 mm, **C**); wire-reinforced tracheal tube (TUORen Medical Equipment Co., Henan, China, **D**)

Soft tracheal tubes, such as reinforced tubes, tend to move along the posterior pharyngeal wall rather than towards the laryngeal inlet, and they are difficult to navigate to the vocal cords without using Magill forceps [8]. A rigid wire tube body of a video stylet bent along the curve of the nasal cavity was reported to help anaesthesiologists complete NTI more easily [9]. However, not all institutions have visual devices. In another study, the use of a stylet resulted in significantly higher first-attempt intubation success among patients with difficult airways undergoing endotracheal intubation compared with the use of a bougie [10]. The tube core (Fig. 1A) in Fig. 1D, consisting of a malleable rigid stylet, is similar to the wire tube body used in the above study [9]. Thus, we hypothesised that a tube core could facilitate NTI without the use of Magill forceps, and the use of a tube core for NTI is a convenient choice.

## Methods

This study protocol was approved by the Research Ethics Board of The First People’s Hospital of Hefei (No. 2019-12). Written informed consent was obtained from all patients, and the study was conducted in accordance with the Declaration of Helsinki.

We excluded patients with the following characteristics from our study: aged < 18 years or > 80 years; a body mass index (BMI) greater than 30 kg/m^2^; a Cormack and Lehane (CL) score of the laryngoscopic view of 3 or 4 [11]; those receiving anticoagulant therapy; those with a history of nasal deformity (e.g. nasal trauma, surgery, obstruction, or polyps); those with maxillofacial cancers; difficulty anticipated in airway management; mentally ill people; and cervical instability. Standard monitoring equipment was used in the operating room, and none of the study subjects was premedicated. All patients were randomised using computer-generated random numbers, and envelopes containing randomisation numbers were divided into 2 groups (*n* = 30 per group) according to the equipment that would be used to guide NTI: the Magill forceps group (Group M) and the tube core group (Group T).

To calculate the sample size, we conducted a pilot study with 10 patients in each group (total, 20 patients). The NTI time was significantly longer in the Magill forceps group than in the tube core group (Group M, 59.8 (5.1) s vs Group T, 53.1 (3.0) s). For this study, the total sample size to achieve 0.95 power and an α-error of 0.05 was 12 patients per group according to G*Power 3.1.9.4 software. Sixty adult patients who were rated as American Society of Anesthesiologists (ASA) class Ι or Π and whose condition required NTI under general anaesthesia were selected.

An otorhinolaryngologist of our hospital who was blinded to the group assignment used a nasal speculum to check for deformities inside the nostrils and to select the smoother nostril. If the patency of both nostrils was equal, NTI was performed in the right nostril [3]. All patients received the same general anaesthesia with 0.3 μg/kg sufentanil and 1.5–2 mg/kg propofol intravenously, followed by the muscle relaxant cisatracurium (0.15 mg/kg). Manual ventilation was performed with 100% oxygen through a facemask for 3 min before intubation. The selected nostril was packed with gauze containing epinephrine to prevent bleeding. Then, 6.5-mm and 6.0-mm wire-reinforced tracheal tubes were used in males and females, respectively (TUORen Medical Equipment Co., Henan, China. Fig. 1B). Anaesthesia was maintained with propofol, remifentanil, sevoflurane and cisatracurium.

**Fig. 2:**
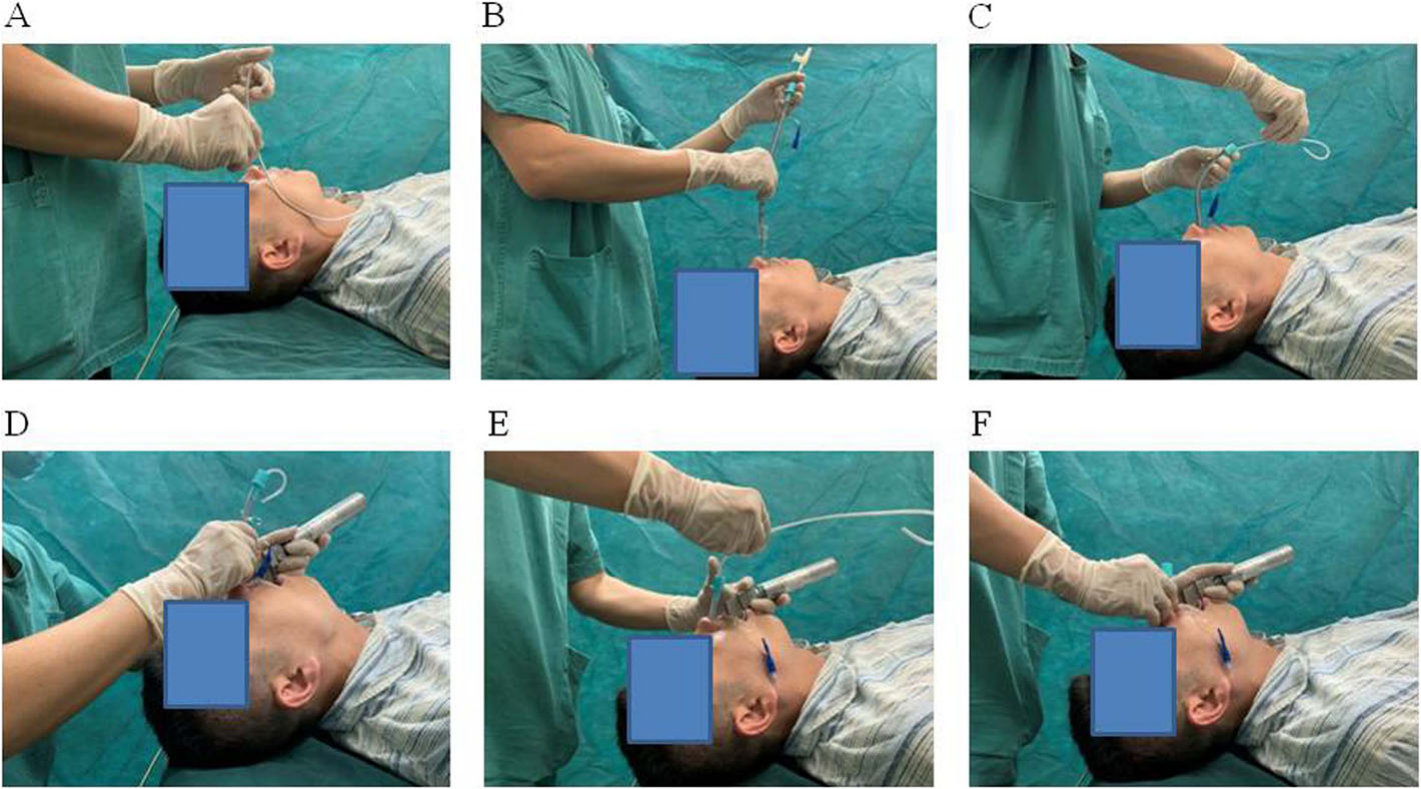
The entire nasotracheal intubation process (**A–F**). **A** The tube core was bent along the curve of the nasal cavity. **B** The tracheal tube was inserted through the nasopharynx under aseptic suction catheter guidance. **C**–**F** Nasotracheal intubation was performed using a tube core after the suction catheter was withdrawn

In Group M, an aseptic suction catheter (outer diameter (OD), 4.0 mm, Fig. 1C) lubricated with liquid paraffin was inserted through the tracheal tube (Fig. 1B) with its tip protruding approximately 15 cm, and the tube was then advanced through the nasopharynx. After that, the suction catheter was removed. A Macintosh laryngoscope was then placed into the patient’s mouth, and the tracheal tube was inserted into the trachea using a conventional technique. However, in Group T, the entire nasotracheal intubation process was performed as presented in Fig. 2A–F: (A) the tube core was bent along the curve of the nasal cavity; (B–C) the tracheal tube was inserted through the nasopharynx under the guidance of a well-lubricated aseptic suction catheter, then the suction catheter was removed before the tube core was inserted into the tracheal tube; and (D–F) the process of nasotracheal intubation was performed using a Macintosh laryngoscope, and then the tube core was withdrawn after the tracheal tube was advanced through the glottis under direct vision. Subsequently, the process of endotracheal intubation was continued, and the tracheal tube was fixed at an appropriate depth. The process was performed by an anaesthesiologist with extensive experience. Minute adjustments to ventilation were performed to maintain the end-tidal carbon dioxide partial pressure at 35–45 mmHg during the entire operation.

The total NTI time, which was defined as the period from when the operator obtained the device to when three successive end-tidal carbon dioxide waves were obtained following intubation [12] was recorded. An observer blinded to the group assignments assessed epistaxis bleeding using direct laryngoscopy five minutes after completing NTI, and bleeding was scored as one of four grades according to the following modified criteria: no epistaxis (no blood observed on either the surface of the tube or the posterior pharyngeal wall); mild epistaxis (blood apparent on the surface of the tube or posterior pharyngeal wall); moderate epistaxis (pooling of blood on the posterior pharyngeal wall); and severe epistaxis (a large amount of blood in the pharynx that impeded NTI and necessitated urgent orotracheal intubation) [13].

After the end of surgery, neostigmine (1 mg) and atropine (0.5 mg) were used to reverse the neuromuscular blockade, and the tracheal tube was extubated when the patient was awake. A visual analogue scale (VAS) score based on a 10-cm vertical scale ranging from 0 = no pain to 10 = worst pain imaginable was recorded by an investigator who was blinded to the group assignments at the following time points: 15 min, 1 h, and 24 h after extubation.

Data are expressed as the mean ± standard deviation. Parametric data were compared between groups by analysis of variance and post hoc testing. Categorical data were analysed using Fisher’s exact test. Statistical significance was considered at *P* values < 0.05. All statistical analyses were performed with Statistical Package for Social Sciences (SPSS) software 20.0.

## Results

**Fig. 3:**
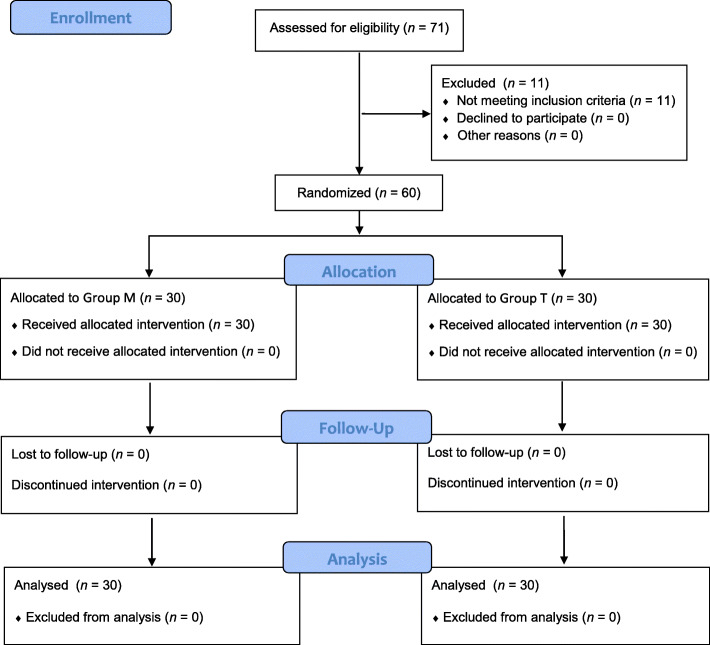
Flow chart illustrating the recruitment and loss of patients in Group M and Group T

In total, 60 patients were enrolled in this study. The CONSORT flow diagram for patient inclusion is shown in Fig. 3. There were no differences between the two groups with regard to demographic data (Table 1).

**Table 1:** Patient characteristics and the duration of anaesthesia

Variable	Group M (*n* = 30)	Group T (*n* = 30)	*P* value
Age (years)	46.4 ± 14.0	49.9 ± 17.7	0.399
Height (cm)	166.3 ± 6.7	166.6 ± 8.7	0.855
Weight (kg)	67.2 ± 11.2	66.5 ± 9.4	0.794
ASA physical status (І/П)	12/18	9/21	0.417
Sex (male:female)	17/13	16/14	0.795
BMI (kg/m^2^)	24.2 ± 3.1	23.9 ± 2.4	0.679
The CL grading scale (1/2)	19/11	17/13	0.598
Duration of anaesthesia (min)	69.2 ± 38.5	85.5 ± 42.1	0.123

Values are expressed as a number or the mean (standard deviation)

There was a significant difference in total NTI time between the two groups (Group M, 59.7 (5.1) s vs Group T, 52.4 (3.1) s) (Table 2).

**Table 2:** NTI time and associated complications

Variable	Group M (*n* = 30)	Group T (*n* = 30)	*P* value
NTI time (s)	59.7 ± 5.1	52.4 ± 3.1	0.000
Epistaxis (mild/moderate/severe)	6 (6/0/0)	5 (5/0/0)	0.739

Values are expressed as a number or the mean (standard deviation)

Mild epistaxis was observed in 6 patients in Group M and 5 patients in Group T (6/30 *vs* 5/30, respectively), and no moderate or severe epistaxis was observed in either group (Table 2). No damage to oral tissue or teeth was observed in either group.

All NTIs were completed successfully. There was no obvious nasal pain at any time point after extubation in either group; therefore, the data are not shown. Additionally, sore throat was not assessed in our study because some of the surgeries were performed on the vocal cords.

## Discussion

Magill forceps are usually needed to facilitate insertion of the endotracheal tube into the glottis [1–3, 12, 14, 15]. However, they may cause rupture of the cuff or mucosal injury and even lead to infection [5–7]. Moreover, this instrument is always not a disposable, sterilised medical device and requires strict aseptic techniques after surgery. The percentage of conventional techniques requiring Magill forceps was reported to be close to 70% [1]; however, this value was 100% in our study. This may be because the wire-reinforced tracheal tube used in our study was so soft that the tips of the tube could not be easily inserted into the glottis. In our present study, a tube core, consisting a disposable sterilised stylet, could completely replace the use of Magill forceps in NTI and significantly decrease the NTI time. This process makes NTI more convenient; furthermore, it may avoid infection caused by Magill forceps, which will be investigated in future research.

Epistaxis, or postpharyngeal bleeding, is the most common complication after NTI. Placement of the stylet in the endotracheal tube first is not recommended in blind NTI because it may cause complications such as bleeding and tissue injury [16]. Sugiyama et al [17] reported that the use of a stylet and a posterior-facing bevel could be implemented in adult patients requiring nasal intubation. Then, it was questioned whether NTI could be associated with traumatic complications that were not confined to the structures within the nose [18]. Thermosoftening of the endotracheal tube should not be overlooked even if other effective methods, such as telescoping the endotracheal tube into a rubber catheter, have already been applied because it has the obvious advantages of reducing the incidence of epistaxis and improving the nasal passage of the endotracheal tube [3, 15]. The wire-reinforced tracheal tube used in our study was a soft endotracheal tube that contributed to reducing bleeding [9]. Therefore, simple thermosoftening of the endotracheal tube was not performed in the present study. However, NTI under suction catheter guidance increases the success rate of airway instrumentation and reduces the incidence and severity of epistaxis [19]. All of these factors may have decreased the severity of epistaxis in our study.

A systematic review demonstrated that the risk of patient infection following the use of a reusable device is significant, warranting the need for stricter guidelines on reprocessing to ensure greater patient safety. Indeed, when considering the risk of infection in the cost analysis, the findings from this study suggest the benefits of disposable medical apparatuses in terms of cost-effectiveness, cross-contamination and resource utilisation [20]. Miller et al [6] found that many cleaning methods could not remove all proteinaceous material, which showed that even following the guidelines for cleaning of equipment may be insufficient to protect patients from transmission of iatrogenic disease, although methods and techniques have been further improved. Staining was even present in 60% of the Magill forceps group. Therefore, this situation prompted us to seriously question the safety of reusable instruments. Perioperative infection has a significant impact on the outcome of surgical patients. Anaesthesiologists play roles in reducing infection by applying appropriate prophylactic measures [21]. Magill forceps are not always disposable sterilised medical devices and require disinfection with strict aseptic techniques after surgery. Perhaps a tube core, consisting of a sterile stylet, should be considered as an optimal choice in NTI when possible. Of course, further study with a large sample is needed to confirm the effectiveness of this instrument.

The wire tube body of the Disposcope endoscope is rigid but can be bent along the curve of the nasal cavity, which has been reported to benefit NTI [9]. Another device, a video intubation stylet for NTI, which has a rigid intubation stylet with an adjustable distal portion, made the NTI process quicker and easier [22]. In those studies, the tube stylet was easily advanced into the glottis by the levelling effect, and the incidence of related complications was not increased [9, 14, 22]. However, not all institutions have visual devices. Although the tube core has a malleable rigid body, it protects the nasal mucosa and the entire nasal passage during the operational process by wire-reinforced tracheal tubes, with results similar to those of a previous study [9].

There are some limitations to our study. First, the operator was not blinded to the study groups, which may have affected the NTI process. Second, the risk of infection was not assessed in our study, which would provide guidance for clinical treatment. We would investigate this aspect in a large clinical trial. Third, patients with preoperative modified Mallampati scores of III or IV were excluded from our study, which should be addressed in future research. Furthermore, NTI was completed under a Macintosh laryngoscope in the present study. However, video laryngoscopy is associated with a significantly decreased force exerted on maxillary incisors and might reduce the risk of dental injury in clinical settings [23]; moreover, video laryngoscopy can be used to clearly observe the glottis, which can simplify the NTI process.

## Conclusions

This study shows that using a tube core, consisting of a disposable sterilised stylet, could completely replace the use of Magill forceps in NTI and significantly reduce the NTI time in patients without a difficult airway. Therefore, the use of a tube core for NTI in patients without a difficult airway may be a convenient choice when possible.

## Data Availability

The datasets used and/or analysed during the current study are available from the corresponding author on reasonable request.

## Abbreviations

NTI: Nasotracheal intubation
BMI: Body mass index
CL: Cormack and Lehane
Group M: Magill forceps group
Group T: Tube core group
ASA: American Society of Anesthesiology
OD: Outer diameter

## References

[1] Lim CW, Min SW, Kim CS, Chang JE, Park JE, Hwang JY (2014). The use of a nasogastric tube to facilitate nasotracheal intubation: a randomised controlled trial. Anaesthesia..

[2] Pourfakhr P, Ahangari A, Etezadi F, Moharari RS, Ahmadi A, Saeedi N (2018). Comparison of nasal intubations by GlideScope with and without a Bougie guide in patients who underwent maxillofacial surgeries: randomized clinical trial. Anesth Analg.

[3] Kim EM, Chung MH, Lee MH, Choi EM, Jun IJ, Yun TH (2019). Is tube Thermosoftening helpful for Videolaryngoscope-guided Nasotracheal intubation?: a randomized controlled trial. Anesth Analg.

[4] Abrons RO, Zimmerman MB, El-Hattab YMS (2017). Nasotracheal intubation over a bougie vs. non-bougie intubation: a prospective randomised, controlled trial in older children and adults using videolaryngoscopy. Anaesthesia..

[5] Nakamura S, Watanabe T, Hiroi E, Sasaki T, Matsumoto N (1997). Hori T: [cuff damage during naso-tracheal intubation for general anesthesia in oral surgery]. Masui..

[6] Miller DM, Youkhana I, Karunaratne WU, Pearce A (2001). Presence of protein deposits on 'cleaned' re-usable anaesthetic equipment. Anaesthesia..

[7] Mahajan R, Batra YK, Kumar S (2007). Another use of Magill forceps to assist nasotracheal intubation. Can J Anaesth.

[8] Kumar R, Gupta E, Kumar S, Rani Sharma K, Rani Gupta N (2013). Cuff inflation-supplemented laryngoscope-guided nasal intubation: a comparison of three endotracheal tubes. Anesth Analg.

[9] Yu J, Hu R, Wu L, Sun P, Zhang Z (2019). A comparison between the Disposcope endoscope and fibreoptic bronchoscope for nasotracheal intubation: a randomized controlled trial. BMC Anesthesiol.

[10] Driver BE, Prekker ME, Klein LR, Reardon RF, Miner JR, Fagerstrom ET (2018). Effect of use of a Bougie vs endotracheal tube and stylet on first-attempt intubation success among patients with difficult airways undergoing emergency intubation: a randomized clinical trial. JAMA..

[11] Cormack RS, Lehane J (1984). Difficult tracheal intubation in obstetrics. Anaesthesia..

[12] Yeom JH, Oh MK, Shin WJ, Ahn DW, Jeon WJ, Cho SY (2017). Randomized comparison of the effectiveness of nasal intubation using a GlideScope video laryngoscope with Magill forceps versus vascular forceps in patients with a normal airway. Can J Anaesth.

[13] Earle R, Shanahan E, Vaghadia H, Sawka A, Tang R (2017). Epistaxis during nasotracheal intubation: a randomized trial of the Parker flex-tip nasal endotracheal tube with a posterior facing bevel versus a standard nasal RAE endotracheal tube. Can J Anaesth.

[14] Staar S, Biesler I, Muller D, Pfortner R, Mohr C, Groeben H (2013). Nasotracheal intubation with three indirect laryngoscopes assisted by standard or modified Magill forceps. Anaesthesia..

[15] Kim YC, Lee SH, Noh GJ, Cho SY, Yeom JH, Shin WJ (2000). Thermosoftening treatment of the nasotracheal tube before intubation can reduce epistaxis and nasal damage. Anesth Analg.

[16] Berry FA (1984). The use of stylet in blind nasotracheal intubation. Anesthesiology..

[17] Sugiyama K, Manabe Y, Kohjitani A (2014). A styletted tracheal tube with a posterior-facing bevel reduces epistaxis during nasal intubation: a randomized trial. Can J Anaesth.

[18] Mahajan R, Nazir R, Gulati S (2014). Mechanism of stylet-facilitated nasotracheal intubation. Can J Anaesth.

[19] Morimoto Y, Sugimura M, Hirose Y, Taki K, Niwa H (2006). Nasotracheal intubation under curve-tipped suction catheter guidance reduces epistaxis. Can J Anaesth.

[20] Mouritsen JM, Ehlers L, Kovaleva J, Ahmad I, El-Boghdadly K (2020). A systematic review and cost effectiveness analysis of reusable vs. single-use flexible bronchoscopes. Anaesthesia..

[21] Shime N (2014). Role of anesthesiologist in prevention of perioperative infection. Masui..

[22] Lee MC, Tseng KY, Shen YC, Lin CH, Hsu CW, Hsu HJ (2016). Nasotracheal intubation in patients with limited mouth opening: a comparison between fibreoptic intubation and the Trachway(R). Anaesthesia..

[23] Schieren M, Kleinschmidt J, Schmutz A, Loop T, Staat M, Gatzweiler KH (2019). Comparison of forces acting on maxillary incisors during tracheal intubation with different laryngoscopy techniques: a blinded manikin study. Anaesthesia..

